# Protective Effects of Nanoceria against Mitochondrial Dysfunction and Angiotensin II-Induced Hypertrophy in H9c2 Cardiomyoblasts

**DOI:** 10.3390/antiox12040877

**Published:** 2023-04-04

**Authors:** Rukhsana Gul, Mushtaq A. Dar, Shahid Nawaz, Assim A. Alfadda

**Affiliations:** 1 Obesity Research Center, College of Medicine, King Saud University, Riyadh 11461, Saudi Arabia; 2 Center of Excellence for Research in Engineering Materials (CEREM), Deanship of Scientific Research (DSR), King Saudi University, Riyadh 11421, Saudi Arabia; 3 Department of Medicine, College of Medicine, King Saud University, P.O. Box 2925, Riyadh 11461, Saudi Arabia

**Keywords:** nanoceria, nano-biomaterial, angiotensin II, hypertrophy, mitochondrial dysfunction, H9c2 cardiomyoblasts

## Abstract

Mitochondrial dysfunction triggered by increased reactive oxygen species (ROS) generation is involved in the pathogenesis and development of cardiac hypertrophy. Nanoceria (cerium oxide nanoparticle) has powerful ROS-scavenging properties and is considered a potential therapeutic option for curbing ROS-related disorders. Here, we explored the signaling mechanism underlying the protective effects of nanoceria against angiotensin (Ang) II-stimulated pathological response in H9c2 cardiomyoblasts. Our data revealed that pretreatment of H9c2 cardiomyoblasts with nanoceria significantly prevented Ang II-stimulated generation of intracellular ROS, aberrant expression of pro-inflammatory cytokines, and hypertrophy markers. Nanoceria pretreatment increased the mRNA levels of genes regulating the cellular antioxidant defense system (SOD2, MnSOD, CAT) in Ang II-treated cells. Furthermore, nanoceria restored mitochondrial function by decreasing mitochondrial ROS, increasing mitochondrial membrane potential (MMP), and promoting the mRNA expression of genes associated with mitochondrial biogenesis (PGC-1α, TFAM, NRF1, and SIRT3) and mitochondrial fusion (MFN2, OPA1). Collectively, these findings demonstrate the protective effects of nanoceria against Ang II-mediated mitochondrial dysfunction and pathological hypertrophy in H9c2 cells.

## 1. Introduction

The disproportionate stimulation of the renin–angiotensin–aldosterone system (RAAS) intensifies the development of cardiovascular diseases by promoting the synthesis of angiotensin II (Ang II) [1,2]. Ang II, upon binding to AT1R, a Gq-protein coupled receptor, rapidly elicits secondary signaling cascades that consequently induce the progression of myocardial hypertrophy [2]. Inhibition of Ang II formation or its binding to the AT1R receptor by ACE inhibitors or ARBs is included in the common therapies for Ang II-stimulated cardiac maladaptive responses such as cardiac hypertrophy [2]. Sustained activation of Ang II results in ROS generation that can contribute significantly to the multiple inflammatory second messenger cytokines [3].

Nanomaterials have noteworthy applications in biomedical research due to their unique physicochemical properties [4,5]. In this perspective, nanoceria has made swift progress in varied biological applications, owing to its capability to switch oxidation states between Ce^3+^ and Ce^4+^, making it a remarkable antioxidant with increased vacancies to capture oxygen [4,5,6,7,8,9,10]. The coexistence of dual oxidation states Ce^3+^ and Ce^4+^ of nanoceria makes it redox-active, hence, allowing for the removal of both superoxide and hydrogen peroxide reliant on the environment. To scavenge reactive oxygen species (ROS) and stop ROS chain reactions, nanoceria mimics both superoxide dismutase (SOD) and catalase (CAT), with the former catalyzing the dismutation of superoxide (O_2_^.−^) to hydrogen peroxide (H_2_O_2_) and molecular oxygen (O_2_), and the later decomposing hydrogen peroxide to oxygen and water [11].

The antioxidant property of nanoceria has supported its potential use in numerous biomedical applications that are largely oxidative-stress-driven, such as inflammation, cancer, neurodegeneration, diabetes, cell apoptosis, and radiation protection of cells [12]. Although, nanoceria’s protective effects against ROS-induced cellular damage have been studied previously, the comprehensive signaling pathway is unclear. To the best of our knowledge, we, for the first time, evaluated nanoceria-mediated protective effects against mitochondrial dysfunction and hypertrophy induced by Ang II. Our data reveal that nanoceria could protect H9c2 cardiomyoblasts against oxidative stress and actuation of proinflammatory and pro-growth signaling stimulated by Ang II. Our data also denoted the positive effects of nanoceria on mitochondrial biogenesis and mitochondrial dynamic alterations in response to Ang II. The findings in the current study demonstrate a potential therapeutic beneficial effect of nanoceria against Ang II-induced pathology, and the results will be helpful for more rational applications of nanoceria in biomedical fields in the future.

## 2. Materials and Methods

### 2.1. Nanoceria Synthesis and Characterization

**Figure 1:**
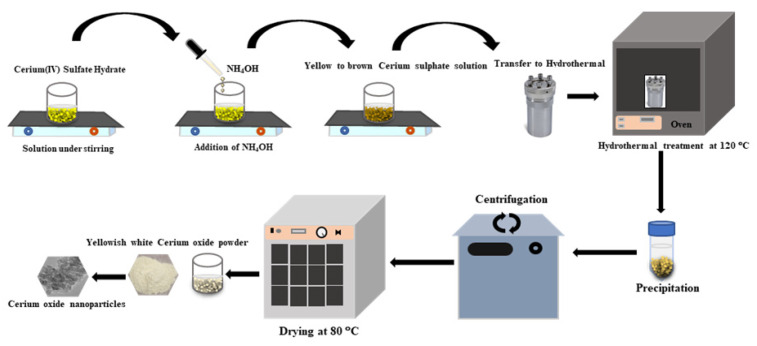
Schematic diagram demonstrating the steps of nanoceria (NC) synthesis using the hydrothermal method.

The protocol for nanoceria preparation via the hydrothermal material fabrication technique has been described previously [13]. In brief, 0.5 M Cerium (III) sulfate hydrate was added to deionized water. After continuous stirring for 10–20 min, ammonium solution was added in a dropwise manner. A Teflon-lined stainless-steel autoclave was used to autoclave the mixture at 120 °C for 5–10 h to react. The autoclave vessels were cooled naturally after the completion of the trial. The resulting product was collected by washing the specimen through a centrifugation process. The precipitated product was dried overnight at 80 °C (Figure 1). The CeO_2_ nanoparticles were characterized by different techniques. The size and morphology of the nanoceria were investigated using transmission electron microscope (TEM, JEM-2100F-JEOL, Tokyo-Japan) and field-emission scanning electron microscope (FESEM, JSM-7600F-JEOL, Tokyo-Japan).

### 2.2. Cell Viability Evaluation

Cell viability was evaluated by MTS assay (Promega, Madison, WI, USA). Cells seeded in 96-well plates overnight were incubated with different concentrations (10, 25, and 50 µg) of nanoceria. After incubation for 72 h, 20 µL of MTS labeling reagent was added to all the wells. Plates were then incubated for 1 h and read at 570 nm using a microplate reader (Biotek Synergy, Winooski, VT, USA).

### 2.3. Cell Culture and Immunoblotting

H9c2 cardiomyoblasts from rat embryonic cardiomyocytes were purchased from (ATCC, CRL-1446). Cells were cultured in complete DMEM (ATCC 30-2002), supplemented with 10% FBS (ATCC, 30-2020), and antibiotic penicillin/streptomycin (100 U/mL: 100 mg/mL) as described previously [14]. Cultures were placed at 37 °C in 5% CO2 air and passaged after 70–80% confluence was achieved. For immunoblotting, H9c2 cardiomyoblasts were treated with nanoceria before Ang II for the desired time period. Cells were trypsinized and lysed to extract proteins as described previously [1,2]. Protein concentration of the lysates was determined by Bradford protein assay (BCA Protein Assay, Pierce, Waltham, MA, USA). For loading, 30–50 μg/lane of lysate was separated by 10% SDS–PAGE and transferred to polyvinylidene difluoride (PVDF) membranes (Bio-Rad Laboratories, Hercules, CA, USA). After transfer, membranes were blocked in 3% bovine serum albumin (BSA) (Sigma) at room temperature for 1 h; blots were incubated overnight at 4 °C with primary antibodies (1:1000 dilution) for phospho-mTOR (pSer2448), mTOR, phospho-p70S6K (pThr389), p70S6K, phospho-RS6 (pSer235/236), RS6, NFkB, SOD2, IL-1β, and iNOS. All antibodies were obtained from Cell Signaling Technology Inc. (Boston, MA, USA). Following primary antibody incubation, membranes were washed 3 times with Tris-buffered saline (TBS)–Tween-20 (TBS-T), incubated with secondary antibodies (peroxidase-conjugated IgG) for 2 h, and washed again 3 times with TBS-T. For stripping and re-probing, membranes were soaked in mild stripping buffer for 20 min and washed several times with TTB-S and re-probed with the target antibody. Chemiluminescence was applied to detect antibody binding to the membranes, and the GeneTools (Syngene, Cambridge, UK) image analysis system was used to obtain images. Quantitation of protein band density was accomplished by using GeneTools image analysis software (Syngene, Cambridge, UK). For NFkB, SOD2, IL-1β, and iNOS, protein band density was normalized to the density of β-actin. For phospho-proteins, results are presented as the ratio between phosphorylated protein and the total protein band intensity using the same membrane.

### 2.4. Intracellular ROS Measurement

Intracellular ROS production was evaluated by employing a cell-permeable redox-sensitive dye 2,7-dichlorodihydrofluorescein diacetate (DCFDA; Molecular Probe, Eugene, OR, USA) as described previously (ROS) [3,14]. After preincubation with DCFDA dye (4 μm) for a period of 30 min, cells were washed two times with DPBS. Nanoceria (10 and 25 µg/mL) was added in media with 2% FBS, and plates were sited at 37 °C for 30 min. After incubation, Ang II was added to all the wells except the control, and fluorescence of DCF was measured at excitation (Ex = 488 nm) and emission (Em = 520 nm) using a 96-well plate reader, Synergy HT multi-mode reader (BioTek Instruments, Inc., Winooski, VT, USA). 

### 2.5. Measurement of Cell Surface Area

H9c2 cardiomyoblasts were plated in 12-well plates and allowed to grow overnight. Cells were pretreated with nanoceria for 30 min before Ang II and incubated at 37 °C in 5% CO_2_ and air for 72 h. After washing twice with DPBS, H9c2 cardiomyoblasts were stained with 300 µL of 0.25% Crystal violet (Sigma Aldrich, Saint Louis, MO, USA) at room temperature for 20 min. Cells were rinsed thrice and random images were obtained by using a ZEISS Axio Observer inverse microscope (Carl Zeiss MicroImaging GmbH, Wetzlar, Germany). Measurement of the cellular surface area (n = 100 cells per group) was carried out by using Zen 2 lite software version 2 (Carl Zeiss Microscopy, Wetzlar, Germany).

### 2.6. RNA Isolation and Quantitative Real-Time Polymerase Chain Reaction

For total RNA isolation from cells, we used TRIzol reagent (Invitrogen, Carlsbad, CA, USA) as per protocol from the manufacturer. Cells were grown in 6-well plates, and 1 mL of TRIzol reagent was added directly to each well. Lysates were transferred to a 1.5 mL tube and 0.2 mL of chloroform was added and mixed thoroughly by shaking. Samples were then centrifuged for 10 min at 12,000× *g* at 4 °C and upper aqueous phase containing the RNA was transferred to new tube. cDNA was synthesized from total RNA (400 ng) by reverse transcription using reverse transcription kits (SuperScript™ III First-Strand Synthesis System, Invitrogen, Waltham, MA, USA). Real-time PCR was performed according to manufacturer instructions using SYBR Green mix (Applied Biosystems, Waltham, MA, USA). Reactions were carried out using real-time PCR (7500 Real-Time PCR System-Thermo Fisher Scientific, Waltham, MA, USA) using forward and reverse primers (Macrogen Inc., Seoul, Republic of Korea) (Table 1). All experiments were carried out in triplicate following thermal cycle conditions: 50 °C-2 min; 95 °C-10 min; 40 cycles of 95 °C-15 s; 60 °C-60 s. β-actin was used as an endogenous control. Target gene expression was quantified to β-actin, and fold changes between treated and untreated groups were calculated by using the 2^−ΔΔCt^ method.

### 2.7. Measurement of Mitochondrial ROS

Mitochondrial ROS was measured by MitoSOX™ Red mitochondrial Superoxide Indicator (Santa Cruz Biotechnology, Dallas, TX, USA) following the manufacturer’s instructions. Cells seeded in 12-well plates were treated with 1 mL of 500 nM of MitoSOX™ Red reagent in HBSS with Calcium and Magnesium. After incubation for 30 min at 37 °C and 5% CO_2_, cells were washed gently with warm PBS and then incubated in HBSS with Calcium and Magnesium. For the detection of mitochondrial ROS, live images were taken within 2 h of staining by cell imaging station (Floid Cell Imaging Solution; Thermo Fisher Scientific, Waltham, MA, USA). The average fluorescence intensity per cell (at least 150 cells/experiment) for each experimental group was analyzed using ImageJ analysis software (National Institutes of Health) [15]. Fluorescence intensity is expressed as fold change vs. control.

### 2.8. Measurement of Mitochondrial Potential

To measure mitochondrial membrane potential, H9c2 cardiomyoblasts were plated in 12-well plates and allowed to grow overnight. After treatment with nanoceria and Ang II for desired times, H9c2 cardiomyoblasts were then treated with 100 nM MitoTracker Green (Applied Biosystems, Inc., Waltham, MA, USA) for 30 min at 37 °C to measure mitochondrial content. After incubation, cells were then co-labeled with 500 nM MitoTracker Red (Applied Biosystems, Inc., Waltham, MA, USA) for 30 min at 37 °C. Cells were incubated at 37 °C for 10 min in Hoechst 33342 (Santa Cruz Biotechnology, Dallas, TX, USA) to stain nuclei. After washing with PBS a couple of times, a complete DMEM was added. Cell images were taken by live-cell imager (Floid Cell Imaging Solution-Thermo Fisher Scientific, Waltham, MA, USA).

### 2.9. Statistical Analysis

Data are presented as means ± standard error of the mean (SEM). The alterations in the different groups were compared by using one-way analysis of variance (ANOVA) followed by Scheffe’s test. Two-group analysis was performed using two-tailed Student’s paired *t*-tests. A value of *p* < 0.05 was considered significant.

## 3. Results 

### 3.1. Characterization of Nanoceria

**Figure 2:**
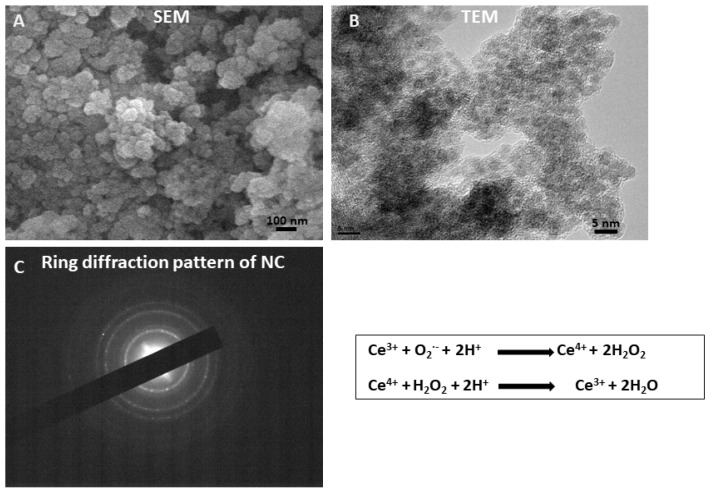
Characterization of NC. (**A**,**B**) represents the SEM (**A**) and TEM images of NC (**B**). (**C**) SAED pattern of NC.

The characterization of synthesized nanoceria was performed by field-emission scanning electron microscopy (FESEM) and transmission electron microscopy (TEM) techniques. Figure 2A is a morphological image obtained via scanning electron microscopy, which reveals the chemical composition of the sample or agglomeration of the nanoparticles, while transmission electron microscopy investigations, i.e., Figure 2B, depict the nature of the synthesized nanoparticles, such as shape, size, and crystallinity. The results disclose the formation of cubic-shaped CeO_2_ nanoparticles with an average particle size of ~3.8 nm, and electron diffraction confirmed the polycrystalline structure of cerium oxide (Figure 2C).

### 3.2. Nanoceria Effects on Cytotoxicity and Ang II-Mediated ROS Generation

**Figure 3:**
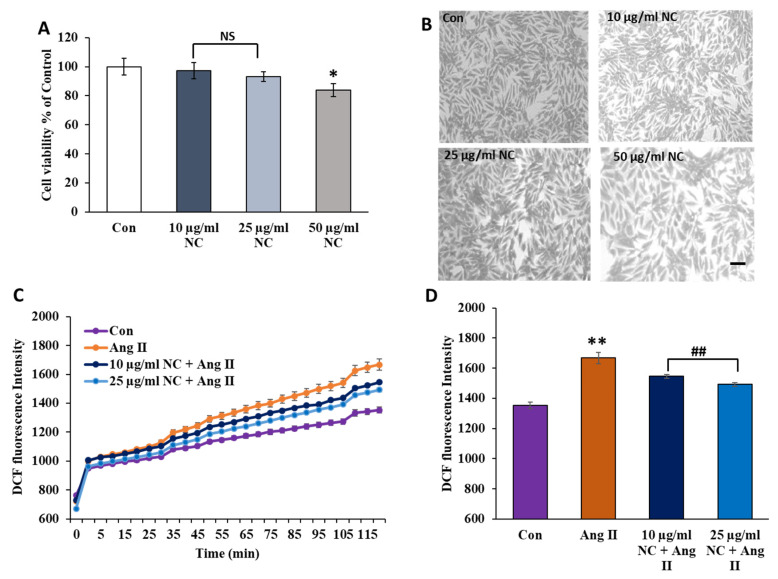
Effects of NC on cell viability and Ang II-induced ROS generation in H9c2 cells. Cell viability was determined by MTS assay following treatment with different concentrations of NC alone. Panel (**A**) shows H9c2 cells treated with 10, 25, and 50 µg/mL NC, respectively. Images of H9c2 cells under a phase-contrast microscope (magnification 10×) using different concentrations of NC (**B**). The morphology of H9c2 cells was not altered by treatment with any concentration of NC (scale bar corresponds to 100 µm). (**C**,**D**) ROS generation was determined by the quantification of DCF fluorescence changes in cells treated with 10 and 25 µg/mL NC prior to Ang II (1 µM) using a microplate reader at an excitation and emission of 488 and 522 nm, respectively. ** *p* < 0.02 vs. Con, * *p* < 0.05 vs. Con, ## *p* < 0.02 vs. Ang II and NS, nonsignificant vs. Con. Values are means ± SEM (n = 6–8 wells for each treatment).

To ascertain the cytotoxic effects of synthesized nanoceria, we examined cell viability by using an MTS assay. H9c2 cells were treated with different concentrations of nanoceria (10, 25, 50 µg/mL) for 72 h (Figure 3A). No significant changes were noticed in cell viability at 10 and 25 µg/mL; however, at 50 µg/mL, a decline of (17 ± 4.4%) was observed compared to the untreated control. Additionally, the morphological examination of H9c2 cardiomyoblasts revealed a normal spindle morphology to the stellate shape of the cell up to 50 µg/mL of nanoceria for 72 h exposure (Figure 3B).

We then determined if ROS generation stimulated by Ang II could be ameliorated by nanoceria in H9C2 cells. Treatment with Ang II increased DCF fluorescence significantly in comparison to untreated control cells. Pretreatment with 10 and 25 µg/mL nanoceria attenuated Ang II-mediated ROS production in these cells (*p* < 0.05, Figure 3C,D). Collectively, these data show that nanoceria has no cytotoxic effects and significantly ameliorates Ang II-mediated oxidative stress. 

### 3.3. Nanoceria Inhibits Ang II-Induced Phosphorylation of Kinases and Protein Expression of Pro-Inflammatory Cytokines

**Figure 4:**
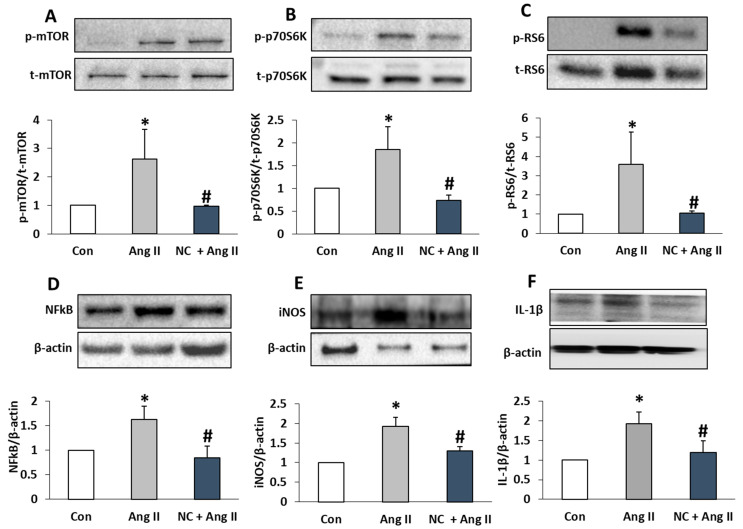
NC inhibited the phosphorylation of the mammalian target of rapamycin complex 1 (mTORC1) cascade and proinflammatory proteins in H9c2 cells. Top: Immunoblots represent phospho/total levels of mTOR (**A**), p70S6K (**B**), and RS6 (**C**) in H9c2 cells treated with Ang II (1 µM) and NC (10 µg/mL) plus Ang II (1 µM). Bottom: Bar graphs represent densitometry analysis of the intensity of the phosphorylated protein bands after adjusting for the intensity of total protein bands. Immunoblots represent protein expression of NFkB (**D**), iNOS (**E**), and IL-1β (**F**) in H9c2 cells treated with Ang II and NC plus Ang II. Bottom: The intensity of the target protein band was normalized to β-actin bands, and densitometry analyses are presented as bar graphs. * *p* < 0.05 vs. Con and # *p* < 0.05 vs. Ang II.

The mammalian target of rapamycin (mTOR) is important for the growth and perpetuation of cardiomyocytes under normal conditions. Overstimulation of mTOR signaling under stress conditions promotes pathological cardiac hypertrophy. Blockade of mTOR has been linked to cardiac protection and a reduction in cardiac hypertrophy. Thus, to understand the useful effects of nanoceria, mTOR and its downstream targets, such as p70S6K and RS6 phosphorylation, were determined in response to Ang II. Ang II stimulated the phosphorylation of mTOR (pSer2448), p70S6K (pThr389), and RS6 (pSer235/236), which was noticeably decreased by pretreatment with nanoceria (Figure 4A–C). An increase in inflammatory markers by Ang II intensifies the development and progression of cardiovascular disease. Thus, we next examined the effect of nanoceria on Ang II-induced protein expression of NFkB, iNOS, and IL-1β markers associated with inflammation. Ang II induced a rise in NFkB, iNOS, and IL-1β protein band intensity, which was diminished by nanoceria pretreatment (Figure 4D–F). Taken together, these findings further signify the positive effects of nanoceria against the detrimental effects of Ang II.

### 3.4. Nanoceria Prevents Hypertrophy and Pro-Inflammatory Gene Expression of Ang II-Induced mRNA Expression of Markers

**Figure 5:**
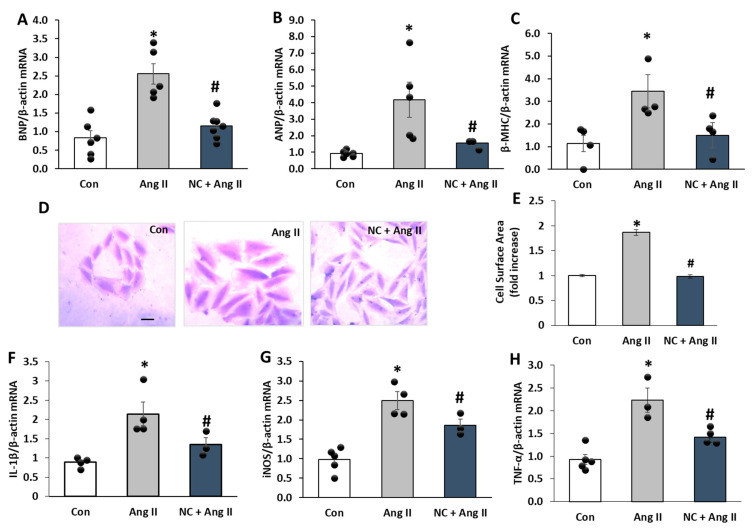
NC reduced the mRNA expression of hypertrophy and proinflammatory genes induced by Ang II in H9c2 cells. Treatment with NC (10 µg/mL) prior to Ang II (1 µM) reduced BNP (**A**), ANP (**B**), and β-MHC (**C**) mRNA expressions compared to Ang II alone. (**D**) Images representing morphological changes in H9c2 cells stained with crystal violet; scale bar = 5 μm. (**E**) Cell surface area was analyzed and is presented as mean (n = 150 cells/group). Treatment with NC prior to Ang II reduced IL-1β (**F**), iNOS (**G**), and TNFα (**H**) mRNA expressions compared to Ang II alone. * *p* < 0.05 vs. Con and # *p* < 0.05 vs. Ang II. Values are means ± SEM (n ≥ 4–6 independent experiments for each treatment group).

To further explore the influence of nanoceria against the deleterious effects of Ang II, we looked at gene expression of cardiac hypertrophy and proinflammatory markers in H9c2 cells. qPCR findings demonstrated that stimulation with Ang II for 72 h augmented the mRNA levels of genes related to cardiac hypertrophy, such as B-type natriuretic peptide (BNP), atrial natriuretic peptide (ANP), and β-myosin heavy chain (MHC), as compared to control. However, pretreatment with nanoceria significantly diminished the mRNA levels of BNP, ANP, and β-MHC triggered by Ang II (Figure 5A–C). We further assessed the effects of nanoceria on Ang II-induced hypertrophy by measuring the changes in cell surface area. Cells treated with Ang II for 72 h showed an increased cell surface area compared to the control, and nanoceria pretreatment significantly reduced it (Figure 5D,E). Furthermore, Ang II-stimulated elevation in mRNA expressions of inflammatory genes, such as TNF-α, IL-1β, and iNOS, was also downregulated by nanoceria pretreatment (Figure 5F–H). Together, these demonstrate that nanoceria plays a protective role against Ang II-induced pro-growth and proinflammatory actions.

### 3.5. Nanoceria Induces Expression of Genes Regulating Antioxidant Defense System

**Figure 6:**
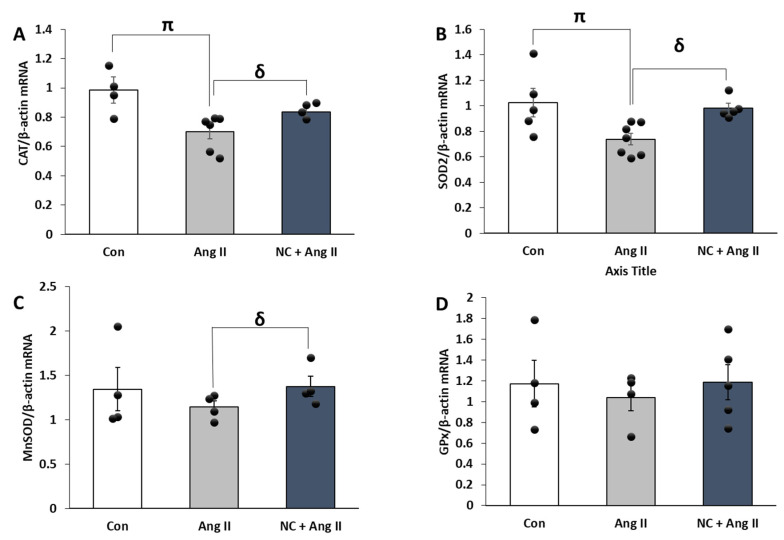
NC increased the expression of genes regulating the antioxidant defense system. mRNA expression levels of hypertrophy genes were examined by RT-qPCR. Ang II decreased mRNA expression of CAT (**A**) and SOD2 (**B**), but not MnSOD (**C**) and GPx (**D**). Treatment with NC (10 µg/mL) prior to Ang II (1 µM) increased SOD2, MnSOD, and CAT mRNA expression compared to Ang II alone. ^π^ *p* < 0.05 vs. Con and ^δ^ *p* < 0.05 vs. Ang II. Values are means ± SEM (n ≥ 4–6 independent experiments for each treatment group).

The cellular antioxidant defense system is important for ROS scavenging. Thus, to assess the influence of nanoceria in triggering the antioxidant defense system to scavenge Ang II-stimulated ROS generation, we determined the gene expression of different antioxidants. qPCR results confirmed a reduction in mRNA levels of superoxide dismutase (SOD)2 and Catalase (CAT) by Ang II, whereas glutathione peroxidase (GPx) and MnSOD were unaffected (Figure 6A–D). Pretreatment with nanoceria significantly increased mRNA levels of SOD2, MnSOD, and CAT compared to Ang II. We also determined the protein expression of SOD2. Ang II treatment showed a decrease in protein expression of SOD2, while pretreatment with nanoceria significantly increased (Supplementary Figure S2). These findings, thus, demonstrate that nanoceria induces stimulation of the cellular antioxidant defense system in H9c2 cardiomyoblasts.

### 3.6. Nanoceria Induces Mitochondrial Biogenesis in Ang II-Treated Cells

**Figure 7:**
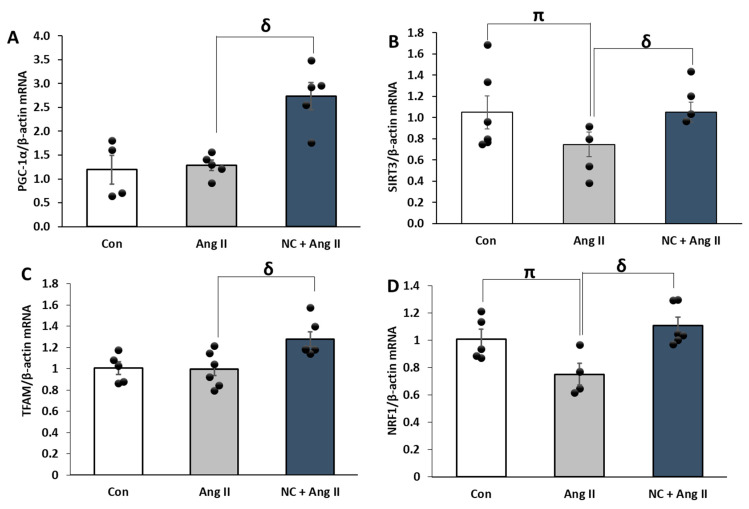
NC decreased the mRNA expression genes regulating mitochondrial biogenesis in H9c2 cells. Treatment with NC (10 µg/mL) prior to Ang II (1 µM) upregulated the gene expression of PGC-1α (**A**), SIRT3 (**B**), TFAM (**C**), and NRF1 (**D**) compared to Ang II alone. ^π^ *p* < 0.05 vs. Con and ^δ^ *p* < 0.05 vs. Ang II. Values are means ± SEM (n ≥ 4–6 independent experiments for each treatment group).

Protective effects of nanoceria were assessed on mitochondrial biogenesis in the presence or absence of Ang II. An increase in the mRNA levels of PPAR-γ coactivator-1α (PGC-1α), sirtuins 3 (SIRT3), mitochondrial transcription factor A (TFAM), and nuclear-related factor 1 (NRF1) was observed in Ang II-stimulated cells pretreated with nanoceria compared to Ang II alone (Figure 7A–D). No significant changes were identified in the mRNA expressions of PGC-1α between untreated control and Ang II, although a reducing trend was perceived in Ang II-treated cells. By contrast, notable differences were apparent between untreated control and Ang II in SIRT3, TFAM, and NRF1 mRNA levels. These findings suggest that nanoceria promotes mitochondrial biogenesis.

### 3.7. Nanoceria Regulates Mitochondrial Dynamics and Mitochondrial Membrane Potential in Ang II-Treated Cells

**Figure 8:**
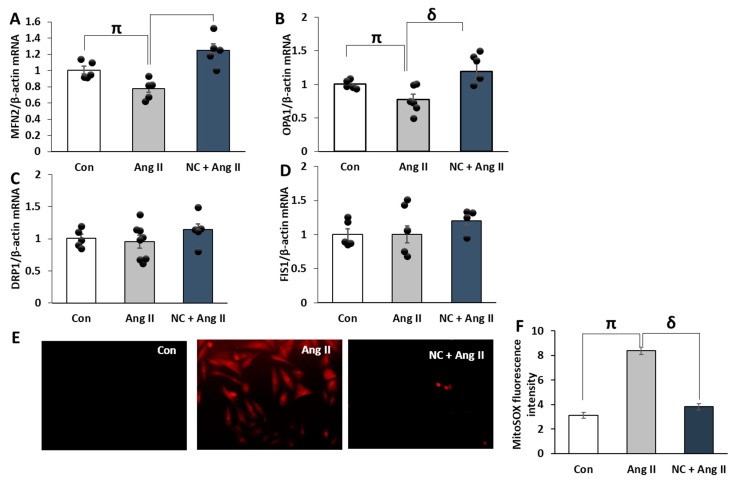
NC changed the mRNA expression of genes regulating mitochondrial dynamics in H9c2 cells. Treatment with NC (10 µg/mL) prior to Ang II (1 µM) upregulated the expression of fusion genes. NC upregulated the mRNA levels of MFN2 (**A**), and OPA1 (**B**) compared to Ang II alone, whereas changes in DRP1 (**C**) and FIS1 (**D**) were not significant. Panel (**E**,**F**) images show mitochondria ROS generation using Mito SOX red stain and relative fluorescent intensity of Mito SOX red (n = 6–8 wells for each treatment). ^π^ *p* < 0.05 vs. Con and ^δ^ *p* < 0.05 vs. Ang II. Values are means ± SEM (n ≥ 4–6 independent experiments for each treatment group).

The fluctuations in mitochondrial dynamics that include fission and fusion maintain the stability of mitochondria and play a pivotal role in cardiovascular disease. A decrease in the content of mitochondria and mitochondrial membrane potential (MMP) is an important characteristic of mitochondrial impairment induced by stimulants. Thus, to evaluate the useful effects of nanoceria on mitochondrial damage induced by Ang II, H9c2 cardiomyoblasts were treated with nanoceria before stimulation with Ang II. We then measured the expression of genes implicated in mitochondrial fusion, such as mitofusin 2 (MFN2), optic atrophy 1 (OPA1), fission dynamin-related protein 1 (DRP1), and fission protein-1 (FIS-1). In contrast to untreated control, treatment with Ang II abated the levels of MFN2 and OPA1, which were remarkably raised by pretreatment with nanoceria. On the contrary, mRNA levels of DRP1 and FIS1 remained unaffected in Ang II-stimulated cells in comparison to untreated control, whereas pretreatment with nanoceria showed an increasing trend in both DRP1 and FIS1 levels (Figure 8A–D). These data further demonstrate that nanoceria incites beneficial effects in Ang II-treated cells by restoring mitochondrial fission- and fusion-associated genes.

The protective effects of nanoceria on Ang II-triggered mitochondrial impairment were evaluated by assessing the MMP in cells. MMP was calculated by finding the ratio of MitoTracker Red (Mito Red) to Mito Tracker Green (MitoGreen). Cells treated with Ang II displayed a lower MMP and decreased content of mitochondria, which was inverted by nanoceria pretreatment (Supplementary Figure S1A,B). The polarized functional mitochondria were significantly lowered in cells stimulated with Ang II compared to untreated control, as reflected by a decrease in MitoRed to MitoGreen in H9c2 cardiomyoblasts. Conversely, pretreatment with nanoceria increased the ratio of MitoRed to MitoGreen in Ang II-treated cells.

Mitochondrial ROS generation is tightly coupled with mitochondrial damage. Thus, to further clarify the role of nanoceria in ameliorating Ang II-mediated mitochondrial dysfunction, we tested nanoceria’s effects on mitochondrial ROS generation. Consistent with our previous observation compared to the control, Ang II treatment in H9C2 cells increased mitochondrial ROS generation, shown by an increase in MitoSOX red fluorescence (Figure 8E,F). However, pretreatment with nanoceria significantly reduced mitochondrial ROS production. Collectively, these data further indicate the protective role of nanoceria in preserving mitochondrial function by decreasing mitochondrial ROS generation and by improving MMP and mitochondrial dynamics.

## 4. Discussion

In this study, we report, for the first time, that nanoceria inhibits Ang II-stimulated proinflammatory and pro-growth signaling cascades involved in pathological cardiomyocyte hypertrophy. Our results suggested that nanoceria markedly ameliorates ROS generation triggered by Ang II and the upregulation of genes that modulate inflammatory and hypertrophy pathways in H9c2 cardiomyoblasts. Additionally, we demonstrated that nanoceria had no distinct cytotoxic effects on H9c2 cells but, indeed, was protective and preserved mitochondrial activity and promoted mitochondrial biogenesis in H9c2 cardiomyoblasts stimulated with Ang II.

Cardiomyocyte hypertrophy is an initial adaptative action to combat pressure or volume overload, which might turn into an irreversible decompensated condition, such as dilated chambers and reduced cardiac functions that ultimately cause heart failure and sudden death [16]. The peptide Ang II is the major precursor of the RAAS system that plays a pivotal function in triggering maladaptive hypertrophic growth via the activation of AT1R [1,2]. Overstimulation of AT1R II triggers several signaling cascades, which, in turn, regulate various pathological effects of Ang II. Ang II triggers intracellular ROS production via the stimulation of membrane-bound NADPH oxidase or by inducing mitochondria to generate ROS [17,18]. Under pathological conditions, Ang II-induced excessive production of superoxide and resulting imbalance in the endogenous antioxidant defense system cause oxidative impairments in the myocardium that, in turn, flare up cardiac remodeling by stimulating inflammation and hypertrophy signaling. Consequently, there is an urgent need to develop and study novel effective inhibitors to prevent and treat the deleterious actions of Ang II.

Nanoceria has recently received attention in different potential applications of biomedicine due to its high biocompatibility and antioxidant benefits [19]. Nanoceria can switch oxidation states between Ce^3+^ and Ce^4+^ on the surface, which plays a critical role in eliminating ROS [18,20]. Additionally, nanoceria can mimic SOD and CAT activity, the antioxidant enzymes that shield the cells against free-radical-induced oxidative damage. The catalytic antioxidant properties of nanoceria have supported their use in therapeutic applications against multiple serious disorders stimulated by oxidative damage [11]. Recent evidence from multiple studies has denoted that nanoceria is cardioprotective against ROS-induced inflammatory response and myocardial reperfusion injury [21,22]. In the animal model of cardiomyopathy, nanoceria administration significantly reduced progressive cardiac ventricular remodeling and dysfunction by inhibiting oxidative damage and inflammatory signaling. Furthermore, cardioprotective effects of nanoceria against Isoproterenol, a β1 adrenergic receptor agonist-induced pro-inflammatory, and profibrotic effects have been demonstrated recently [21]. However, according to our information, no studies so far have investigated the cardioprotective effects of nanoceria against Ang II stimulation. Our current findings indicate that nanoceria significantly inhibits Ang II-induced upregulation in ROS production and phosphorylation of mTOR, p70S6K, and RS6 at pSer2448, pThr389, and pSer235/236, respectively. Furthermore, to understand the effects of nanoceria on mechanisms underlying the pathophysiological actions of Ang II, the expression of genes regulating hypertrophy and inflammatory signaling in cells was studied. Interestingly, nanoceria significantly diminished the expression of genes related to cardiac hypertrophy (BNP, ANP, and β-MHC) and pro-inflammatory signaling (IL-1β, TNF-α, and iNOS) in H9c2 cells. In agreement with the previous findings, nanoceria increased the expression of genes regulating the intracellular antioxidant defense system. Ang II stimulation reduced the levels of antioxidant genes SOD2 and CAT compared to untreated control. However, pretreatment with nanoceria markedly increased SOD2, MnSOD, and CAT gene expression in Ang II-treated cells.

Mitochondria are well known to generate ROS and, under physiological conditions, ROS production is balanced by an intracellular antioxidant defense system involving mitochondrial enzymes MnSOD and GPx. However, pathological conditions deplete antioxidant activity causing oxidative damage that, in turn, aggravates structural and functional abnormalities in mitochondria [23]. These abnormalities in mitochondria are termed mitochondrial dysfunction that, in turn, is attributed to decompensated cardiac failure [24,25,26]. Given the relationship between the progression of heart failure to mitochondrial dysfunction, mitochondrial biogenesis to increase the number of healthy mitochondria is crucial for new therapeutic targets. Mitochondrial biogenesis is mainly controlled by PGC-1α, a co-transcriptional factor that enhances the activity of NRF1 to alleviate TFAM expression. TFAM stimulates mitochondrial biogenesis by increasing the transcription and replication of mitochondrial DNA [27]. An NAD+-dependent deacetylase and highly conserved member of the sirtuins family, SIRT3 plays a vital function in mitochondrial biogenesis by promoting PGC-1α in addition to maintaining cellular energy homeostasis [28]. Studies have demonstrated the association between impaired mitochondrial biogenesis and deleterious cardiac diseases, particularly in cardiomyocyte hypertrophy and failure after stimulation with different agents. In the present study, the cardiac protective effects of nanoceria against Ang II stimulation were further demonstrated by increased expression of genes involved in mitochondrial biogenesis (PGC-1α, NRF1, TFAM, and SIRT3). 

Mitochondrial homeostasis is tightly regulated by mitochondrial dynamics, which refer to a constant balance of fusion and fission processes. Under stress situations, an increase in mitochondrial ROS production leads to reduced MMP, decreased mitochondrial content, and an imbalance in fission and fusion processes. These perturbations in mitochondrial dynamics in various pathological conditions lead to the accumulation of malfunctioning mitochondria [29]. Accumulating evidence suggests that aberrations in mitochondrial dynamics triggered by increased mitochondrial ROS production accelerate the progression of cardiovascular disease. Consequently, we studied the effects of nanoceria on mitochondrial ROS generation and the expression of genes related to fusion and fission in H9c2 cardiomyoblasts. Treatment with Ang II increased mitochondrial ROS generation compared to control, which was reduced by NC pretreatment. Furthermore, Ang II reduced mitochondrial fusion-related genes, including MFN2 and OPA1, compared to untreated control. However, the mRNA levels of genes regulating mitochondrial fission, such as FIS1 and DRP1, remained unchanged after Ang II treatment. Interestingly, in the nanoceria pretreated group, MFN2 and OPA1 gene expressions were significantly increased in comparison to the Ang II-treated group. On the contrary, DRP1 and FIS1 levels were not significantly different in the nanoceria pretreated group compared to Ang II alone. These results illustrate that nanoceria protects mitochondria by restoring mitochondrial dynamics via elevating mitochondrial fission in Ang II-treated H9c2 cardiomyoblasts.

Previous studies have illustrated that a decreased mitochondrial membrane potential is tightly linked to the loss of mitochondrial function. Thus, to determine mitochondrial function, we examined nanoceria-mediated changes in MMP by using MitoRed in H9c2 cardiomyoblasts in response to Ang II. Total mitochondrial content was determined using MitoGreen. Ang II-stimulated cells exhibited a lower MMP and decreased total mitochondrial content; however, cells incubated with nanoceria before Ang II displayed an elevated MMP and total mitochondrial content. The ratio of MitoRed to MitoGreen, which reflects levels of polarized functional mitochondria, was lower in Ang II-treated cells than in controls. Pretreatment with nanoceria increased the ratio of MitoRed to MitoGreen in the H9c2 cardiomyoblasts, which suggested that nanoceria could preserve the mitochondrial function upon Ang II stimulation.

## 5. Conclusions

**Figure 9:**
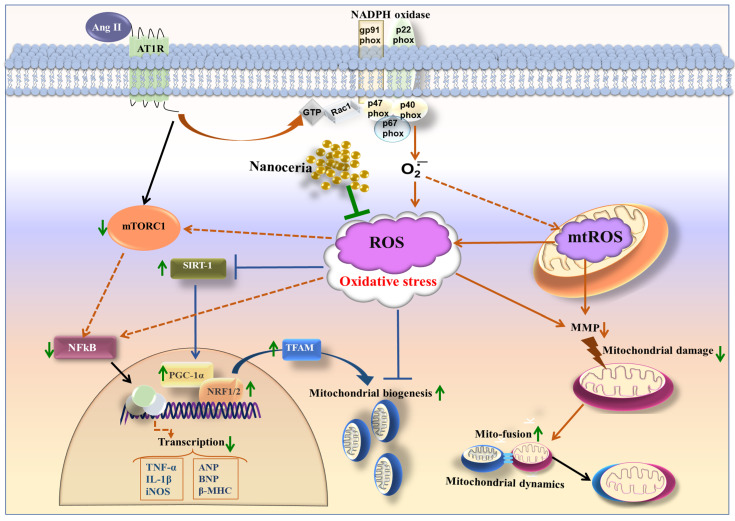
Schematic diagram depicting the protective effects of NC (arrows in green) on Ang II-induced signaling in H9c2 cardiomyoblasts.

In conclusion, the current study demonstrated the protective effects of nanoceria against Ang II-triggered oxidative damage and mRNA levels of genes regulating proinflammatory and hypertrophy signaling. Treatment of H9c2 cells with nanoceria reduced ROS production and enhanced the expressions of genes (SOD2, MnSOD, CAT) regulating the cellular antioxidant defense system. Furthermore, nanoceria increased MMP and enhanced genes regulating mitochondrial biogenesis (PGC-1α, SIRT3, NRF1, and TFAM) and mitochondrial fusion (MFN2, OPA1) (Figure 9). These findings demonstrate that nanoceria alleviates Ang II-induced mitochondrial dysfunction by augmenting mitochondrial biogenesis and dynamics. 

## Figures and Tables

**Table 1:** Primer Sequences.

Gene	Forward Primer	Reverse Primer	Accession Number
ANP	AAAGCAAACTGAGGGCTCTGCTCG	TTCGGTACCGGAAGCTGTTGCA	XM_032887567.1
BNP	ACAATCCACGATGCAGAAGC	CGCCGATCCGGTCTATCTTC	XM_032887581.1 XM_032887581
β-MHC	ATCTACAGCGGGTGAAGCAG	CAGGTTAGCCTTGGCCTTGA	NM_017240.2 NM_017240.2
TNF-α	CACTCAGGCATCGACATTCG	CACCGGCAAGGATTCCAA	XM_032888689.1
iNOS	CGGCCACCAGCTTCTTCA	TGCTTACAGGTCTACGTTCAAGACAT	XM_032912147.1
IL-1β	AGGCCACAGGTATTTTGTCG	GACCTTCCAGGATGAGGACA	NM_031512.2
SIRT3	ACATACGGGCTGACGTGATG	AGTCGGGGCACTGATTTCTG	XM_032891512.1
PGC-1α	ACTCAGCAAGTCCTCAGTGC	TTCTGGTGCTGCAAGGAGAG	NM_031347.1
TFAM	TGTCATTGGGATTGGGCACA	AGATGCACGCACAGTCTTGA	XM_032888687.1
NRF1	CATGGCCCTTAACAGTGAAGC	TGGTCCATGCATGAACTCCA	NM_001100708.1
MFN2	TGTTCAGAGGCCATCGGTTC	TCCACCTGTCTGAACTTCACC	XM_008764288.3
FIS1	GGGTTACATGGATGCCCAGA	TTTGGGCAACAGCTCCTCC	XM_032886584.1
DRP1	ACAACAGGAGAAGAAAATGGAGTTG	TGGATTGGCTCAGGGCTTAC	NM_053655.3
OPA1	TCTTCACTGCGGGTACACCT	TCCTTCTCCAAACGCTCCAG	XM_017597866.2
MnSOD	ACCACAGGCCTTATTCCACT	TACAACAGCTCAGCCACAGT	Y00497.1
CAT	TCCCAGAAGCCTAAGAATGCA	GCGATGATTACTGGTGAGGCT	NM_012520.2
GPx	CAGTCCACCGTGTATGCCTT	TGCCATTCTCCTGATGTCCG	NM_030826.4
SOD-2	ACCACAGGCCTTATTCCACT	TACAACAGCTCAGCCACAGT	XM_032894729.1
β-actin	CAACGTCACACTTCATGATGGA	ATGCCCCGAGGCTCTCTT	XM_032887061.1

## Data Availability

Data is contained within the article and supplementary material.

## Supplementary Materials

The following supporting information can be downloaded at: https://www.mdpi.com/article/10.3390/antiox12040877/s1, Figure S1: NC effect on mitochondrial membrane potential in Ang II-induced mitochondrial ROS generation in H9c2 cells. Figure S2: Effect of NC on SOD2 protein expression in H9c2 cells.

## References

[1] Gul R., Kim S.Y., Park K.H., Kim B.J., Kim S.J., Im M.J., Kim U.H. (2008). A novel signaling pathway of ADP-ribosyl cyclase activation by angiotensin II in adult rat cardiomyocytes. Am. J. Physiol. Heart Circ. Physiol..

[2] Gul R., Park J.H., Kim S.Y., Jang K.Y., Chae J.K., Ko J.K., Kim U.H. (2009). Inhibition of ADP-ribosyl cyclase attenuates angiotensin II-induced cardiac hypertrophy. Cardiovasc. Res..

[3] Gul R., Shawl A.I., Kim S.H., Kim U.H. (2012). Cooperative interaction between reactive oxygen species and Ca^2+^ signals contributes to angiotensin II-induced hypertrophy in adult rat cardiomyocytes. Am. J. Physiol. Heart Circ. Physiol..

[4] Saifi M.A., Seal S., Godugu C. (2021). Nanoceria, the versatile nanoparticles: Promising biomedical applications. J. Control Rel..

[5] Thakur N., Manna P., Das J. (2019). Synthesis and biomedical applications of nanoceria, a redox active nanoparticle. J. Nanobiotechnol..

[6] Celardo I., De Nicola M., Mandoli C., Pedersen J.Z., Traversa E., Ghibelli L. (2011). Ce³^+^ ions determine redox-dependent anti-apoptotic effect of cerium oxide nanoparticles. ACS Nano.

[7] Dunnick K.M., Pillai R., Pisane K.L., Stefaniak A.B., Sabolsky E.M., Leonard S.S. (2015). The Effect of Cerium Oxide Nanoparticle Valence State on Reactive Oxygen Species and Toxicity. Biol. Trace Elem. Res..

[8] Kim J., Hong G., Mazaleuskaya L., Hsu J.C., Rosario-Berrios D.N., Grosser T., Cho-Park P.F., Cormode D.P. (2021). Ultrasmall Antioxidant Cerium Oxide Nanoparticles for Regulation of Acute Inflammation. ACS Appl. Mater. Interfaces.

[9] Nelson B.C., Johnson M.E., Walker M.L., Riley K.R., Sims C.M. (2016). Antioxidant Cerium Oxide Nanoparticles in Biology and Medicine. Antioxidants.

[10] Niu J., Wang K., Kolattukudy P.E. (2011). Cerium oxide nanoparticles inhibit oxidative stress and nuclear factor-κB activation in H9c2 cardiomyocytes exposed to cigarette smoke extract. J. Pharmacol. Exp. Ther..

[11] Baldim V., Bedioui F., Mignet N., Margaill I., Berret J.F. (2018). The enzyme-like catalytic activity of cerium oxide nanoparticles and its dependency on Ce(3+) surface area concentration. Nanoscale.

[12] Stephen Inbaraj B., Chen B.H. (2020). An overview of recent in vivo biological application of cerium oxide nanoparticles. Asian J. Pharm. Sci..

[13] Dar M., Gul R., Alfadda A., Karim M., Kim D.-W., Cheung C., Almajid A.A., Alharthi N.H., Pulakat L. (2017). Size-dependent effect of nanoceria on their antibacterial activity towards Escherichia coli. Sci. Adv. Mater..

[14] Gul R., Alsalman N., Bazighifan A., Alfadda A.A. (2021). Comparative beneficial effects of nebivolol and nebivolol/valsartan combination against mitochondrial dysfunction in angiotensin II-induced pathology in H9c2 cardiomyoblasts. J. Pharm. Pharmacol..

[15] Schneider C.A., Rasband W.S., Eliceiri K.W. (2012). NIH Image to ImageJ: 25 years of image analysis. Nat. Methods.

[16] Messerli F.H., Rimoldi S.F., Bangalore S. (2017). The Transition From Hypertension to Heart Failure: Contemporary Update. JACC Heart Fail..

[17] Dikalov S.I., Nazarewicz R.R. (2013). Angiotensin II-induced production of mitochondrial reactive oxygen species: Potential mechanisms and relevance for cardiovascular disease. Antioxid. Redox Signal..

[18] Chen J., Patil S., Seal S., McGinnis J.F. (2006). Rare earth nanoparticles prevent retinal degeneration induced by intracellular peroxides. Nat. Nanotechnol..

[19] Casals E., Zeng M., Parra-Robert M., Fernández-Varo G., Morales-Ruiz M., Jiménez W., Puntes V., Casals G. (2020). Cerium Oxide Nanoparticles: Advances in Biodistribution, Toxicity, and Preclinical Exploration. Small.

[20] Karakoti A.S., Monteiro-Riviere N.A., Aggarwal R., Davis J.P., Narayan R.J., Self W.T., McGinnis J., Seal S. (2008). Nanoceria as Antioxidant: Synthesis and Biomedical Applications. JOM (1989).

[21] Kumari P., Saifi M.A., Khurana A., Godugu C. (2018). Cardioprotective effects of nanoceria in a murine model of cardiac remodeling. J. Trace Elem. Med. Biol..

[22] Sangomla S., Saifi M.A., Khurana A., Godugu C. (2018). Nanoceria ameliorates doxorubicin induced cardiotoxicity: Possible mitigation via reduction of oxidative stress and inflammation. J. Trace Elem. Med. Biol..

[23] Hollensworth S.B., Shen C., Sim J.E., Spitz D.R., Wilson G.L., LeDoux S.P. (2000). Glial cell type-specific responses to menadione-induced oxidative stress. Free Radic. Biol. Med..

[24] Yancey D.M., Guichard J.L., Ahmed M.I., Zhou L., Murphy M.P., Johnson M.S., Benavides G.A., Collawn J., Darley-Usmar V., Dell’Italia L.J. (2015). Cardiomyocyte mitochondrial oxidative stress and cytoskeletal breakdown in the heart with a primary volume overload. Am. J. Physiol. Heart Circ. Physiol..

[25] Yuan M., Gong M., Zhang Z., Meng L., Tse G., Zhao Y., Bao Q., Zhang Y., Yuan M., Liu X. (2020). Hyperglycemia Induces Endoplasmic Reticulum Stress in Atrial Cardiomyocytes, and Mitofusin-2 Downregulation Prevents Mitochondrial Dysfunction and Subsequent Cell Death. Oxid. Med. Cell. Longev..

[26] Jiang X., Cai S., Jin Y., Wu F., He J., Wu X., Tan Y., Wang Y. (2021). Irisin Attenuates Oxidative Stress, Mitochondrial Dysfunction, and Apoptosis in the H9C2 Cellular Model of Septic Cardiomyopathy through Augmenting Fundc1-Dependent Mitophagy. Oxid. Med. Cell. Longev..

[27] Gureev A.P., Shaforostova E.A., Popov V.N. (2019). Regulation of Mitochondrial Biogenesis as a Way for Active Longevity: Interaction Between the Nrf2 and PGC-1α Signaling Pathways. Front. Genet..

[28] Pillai V.B., Sundaresan N.R., Jeevanandam V., Gupta M.P. (2010). Mitochondrial SIRT3 and heart disease. Cardiovasc. Res..

[29] Lee Y.J., Jeong S.Y., Karbowski M., Smith C.L., Youle R.J. (2004). Roles of the mammalian mitochondrial fission and fusion mediators Fis1, Drp1, and Opa1 in apoptosis. Mol. Biol. Cell.

