# Supplementary material for: Protective Effects of Nanoceria against Mitochondrial Dysfunction and Angiotensin II-Induced Hypertrophy in H9c2 Cardiomyoblasts
Source: Antioxidants (Basel). 2023 Apr 4;12(4):877. doi: 10.3390/antiox12040877 (PMC10135342; doi:10.3390/antiox12040877)
Supplement: Supplementary file 1 [file antioxidants-12-00877-s001.zip › antioxidants-2232590-supplementary.pdf]

Supplementary Fig S1

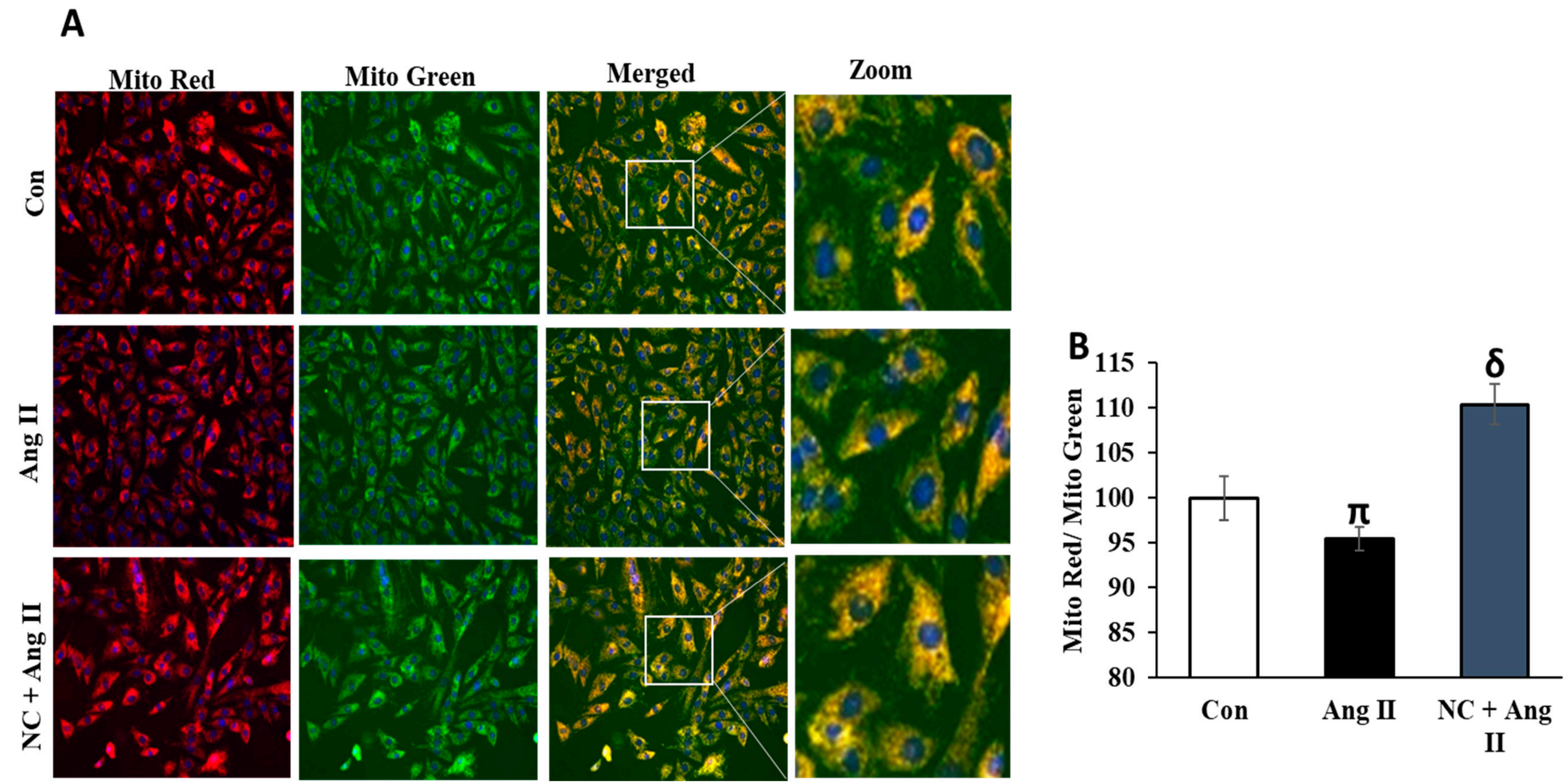

Supplementary Figure S1. NC effect on mitochondrial membrane potential in Ang II-induced mitochondrial ROS generation in H9c2 cells

Panel A displays the representative micrograph of Mito Tracker Red and Mito Tracker Green and Hoechst 33342 stained H9c2 cells showing MMP. Panel B shows the relative fluorescent intensity of Mito Tracker Green (B), Mito Tracker red (C), and the relative fluorescent intensity of Mito Tracker red normalized to the relative fluorescence intensities of MitoTracker green (n = 4 wells for each treatment) (D). $^{\pi}P < 0.05$ vs. untreated (Con), $^{\delta}P < 0.05$ vs. Ang II and NS, nonsignificant; $^{\rho}P < 0.05$ vs. Neb + Ang II. Values are means ± SEM. N ≥ 6 for each treatment group.

Supplementary Fig S2.

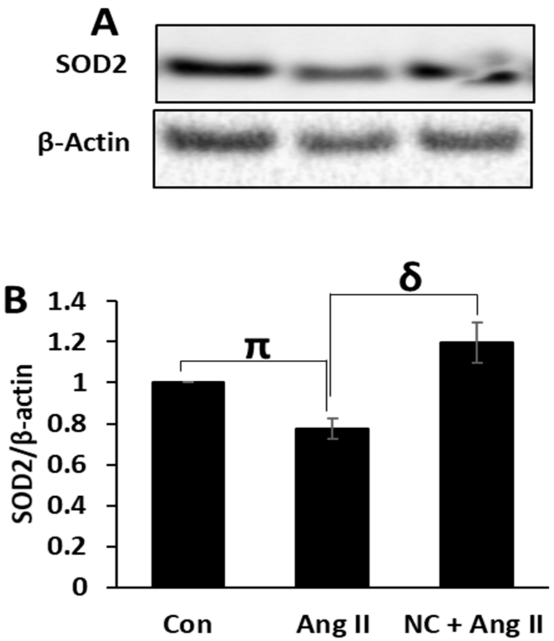

Supplementary Figure S2. Effect of NC on SOD2 protein expression in H9c2 cells. Top: Immunoblot represent SOD2 protein expression in H9c2 cells treated with Ang II (1 µM) and NC (10 µg/ml) plus Ang II (1 µM). Bottom: Bar graphs represent densitometry analysis of the intensity of the protein bands after adjusting for the intensity of β-actin. $^{\pi}P < 0.05$ vs. Con and $^{\delta}P < 0.05$ vs. Ang II.
